# Effect of Larval Nutritional Regimes on Morphometry and Vectorial Capacity of *Aedes aegypti* for Dengue Transmission

**DOI:** 10.1155/2019/3607342

**Published:** 2019-10-09

**Authors:** Nayana Gunathilaka, Hasini Upulika, Lahiru Udayanga, Deepika Amarasinghe

**Affiliations:** ^1^Department of Parasitology, Faculty of Medicine, University of Kelaniya, Ragama, Sri Lanka; ^2^Department of Zoology and Environment Management, Faculty of Science, University of Kelaniya, Dalugama, Sri Lanka; ^3^Department of Bio-systems Engineering, Faculty of Agriculture and Plantation Management, Wayamba University of Sri Lanka, Makandura, Sri Lanka

## Abstract

**Background:**

Nutritional level in larval diet of mosquito vectors influence on life history traits and vectorial capacity (VC). Therefore, the present study was carried out to assess the effect of larval diet concentration on vector bionomic and VC of *Aedes aegypti* in Sri Lanka.

**Method:**

Three batches of 400 *Ae*. *aegypti* larvae (first instar) were reared under different concentrations of larval diet (6%, 8%, and 10%; Volume/Volume), which was prepared by mixing 12.5 g of tuna meal, 9.0 g of bovine liver powder, and 3.5 g of Brewer's yeast, in 100 ml of distilled water. The effect of larval diet concentration on different morphometric and functional parameters of larvae (length and width of head, abdomen, survival rate, and pupation success), pupae (length and width of cephalothorax, survival rate, and adult emersion), adult (length and width of thorax, abdomen, survival rate, longevity, biting frequency and fecundity of adults) were examined. In addition, VC of *Ae*. *aegypti* was evaluated. The General Linear Model (GLM) was used for the statistical analysis.

**Results:**

Larval head length, head width, thoracic width, abdominal length, abdominal width, total length, and survival rate significantly increased with higher doses of larval diet (*P* < 0.05). In case of pupae, length, and width of cephalothorax, survival rate and adult emergence rate denoted an increasing trend with the elevated larval diets. However, the variations of survival rate and adult emergence rate were statistically significant (*P* < 0.05). In adults, all morphometric parameters (thoracic length, abdominal length, abdominal width, and wing length) significantly increased with elevating larval diets levels (except for thoracic width) along with the biting frequency, fecundity, and survival rate (*P* < 0.05) of adult females. The VC also denoted significant variations (*F*_4,14_ = 24.048; *P* < 0.05) with the larval diet concentration, whereby the highest VC of 196.37 was observed at 10% treatment.

**Conclusion:**

Larval food availability has a significant influence on the adult fitness and thus may affect the incidence of dengue due to variations in the VC of *Ae*.

## 1. Introduction

Mosquitoes (Diptera; Culicidae) are one of the major threats for millions of people as they act as vectors of large number of infections of pathogens and parasites, namely, malaria, filariasis, and viral infections such as dengue, encephalitis, and yellow fever [1]. *Aedes aegypti*, an invasive species that has been identified in six continents, is frequently dispersed by human activities and represents a serious threat to public health, due to its ability to transmit numerous vector-borne diseases such as urban yellow fever (African and American tropics), dengue, zika, and chikungunya throughout most of the tropical world [2]. It has now become the most widespread mosquito species globally [3–6].

Dengue is a rapidly emerging arthropod-borne virus which is transmitted to humans mainly by *Ae*. *aegypti* and *Ae*. *albopictus* [7]. In Sri Lanka, dengue has been notified as one of the major public health concerns in recent years recording the highest ever cases of dengue in 2017 denoting 186,00 cases from the country and 51,584 cases in 2018 [8].

As there is no effective vaccine or antiviral treatment available up to now for dengue, current dengue prevention efforts are mainly driven on vector control strategies which integrate the use of insecticides with natural enemies, elimination of breeding sites, and physical approaches [9, 10]. However, reduction of dengue incidence through vector control still remains as a challenge in Sri Lanka, despite of continuous control efforts, due to limited knowledge and understanding on the ecological factors that regulate dengue transmission and their relative importance in disease prevention and management [10].

*Ae*. *aegypti* larvae utilize a large variety of habitats enriched with different food types including microorganisms, detritus, biofilm, and other organic matter such as dead invertebrates, plant materials that has been degraded by fungi or bacteria [11]. Further, *Ae*. *aegypti* larvae use a variety of feeding methods such as collecting-gathering, scraping, shredding, and predation [11]. *Ae*. *aegypti* larvae require a diet comprising glucose, salts, lipids, RNA, 17 amino acids, glutathione, and 12 vitamins. Larvae of *Ae*. *aegypti* can survive with a chemically defined diet consisting of glucose, salt mixture, lipids, RNA, 17 amino acids, glutathione, and 12 vitamins. It has been investigated that valine, l-leucine, l-*iso*leucine, l-phenylalanine, l-histidine, l-arginine, l-tryptophane, l-threonine, l-methionine, and l-lysine essential are for larval growth. In addition, l-cystine is essential for pupation and l-proline, l-hydroxyproline, and l-serine are required for normal growth and development. Therefore, the larval diet should be enriched with amino acids. The absence of vitamin B_12_ delays pupation and *p*-aminobenzoic acid interruptions ecdysis [12].

Many studies have highlighted that the residing environment of larval stages of mosquitoes strongly determines the adult characteristics such as individual size, teneral reserves, biting behavior, fecundity, longevity, and vector competence, which affect the overall vectorial capacity [13–16]. Any change in the environment that affects the related aspect of vector biology may result in a variation in risk of disease transmission via influencing the vectorial capacity [17]. At present, many investigations that evaluate the effect of environment on mosquito biology and aspects of Vectorial Capacity (VC) have directly focused on adult mosquitoes, with limited attention on larval stages.

There have been epidemiological models and mathematical based approaches to determine the VC of disease vectors. However, such approaches have not been evaluated for mosquito vectors in Sri Lanka under field or laboratory conditions. Therefore, objective of the present study was to explore the VC of *Ae*. *aegypti,* which is the main vector for dengue transmission in Sri Lanka under laboratory settings at different larval rearing conditions using mathematical based interpretation.

## 2. Method

### 2.1. Establishment of an *Ae*. *aegypti* Mosquito Colony for the Experiment

#### 2.1.1. Rearing of Immature Stages

An *Ae*. *aegypti* colony, maintained at the Department of Parasitology, Faculty of Medicine, University of Kelaniya, Sri Lanka, that has been established from eggs collected from an ovitrap surveillance conducted within the Ragama Medical Officer of Health area was used to collect the eggs for the current study. Laboratory reared colony after 5 generations, which was adapted to laboratory conditions were taken for the study. Eggs sheets were soaked in deoxygenated distilled water and kept for 1-hour duration at room temperature (25°C–28°C). The first instar larvae were collected after 1 hour using a pasture pipette. Larvae were transferred into larval rearing trays (25 × 25 × 7 cm) and reared with a daily dose of larval diet comprising 12.5 g of tuna meal, 9.0 g of bovine liver powder, and 3.5 g of brewer's yeast dissolved in 100 ml of distilled water. Pupae were sorted daily for larval trays and transferred into pupal rearing cups (50 ml capacity) using a pasture pipette [18, 19].

#### 2.1.2. Rearing of Adult *Ae*. *aegypti*

Pupal rearing cups were kept inside adult mosquito rearing cages (30 × 30 × 30 cm) until the emergence of adults. Newly emerged adult mosquitoes were provided with 10% sugar solution and vitamin complex (1 ml). After 24 hours of initial sugar feeding, the adult female mosquitoes were allowed to feed on human blood using an artificial metal plate membrane feeding system [19]. Human blood was collected from a healthy volunteer (<18 years) under sterile conditions by a trained technical officer under supervision of a Medical Officer at the Faculty of Medicine, University of Kelaniya, Sri Lanka.

The written consent from the donor was obtained prior collection of blood. Blood fed females were maintained under a 12 : 12 (light : dark) cycle. The adult rearing cages were covered with wet towels to maintain the humidity at 75±5% and the temperature was maintained at 27±2°C. Sugar and vitamin syrup were kept available for adult mosquitoes. Oviposition cups (diameter 9.5 cm, height 5 cm) lined with a strip of white colored filter paper was kept inside the adult cages three days after the initial blood meal. Eggs deposited on the lined filter paper were used to maintain the mosquito colony throughout the experimental trials.

### 2.2. Influence of Larval Food Quantity on Survival and Development

#### 2.2.1. Larval Feeding Experiment

Three batches of 400 first instar larvae (L1) of *Ae*. *aegypti* were counted and transferred in to three separated larval rearing trays (25 × 25 × 7 cm) containing 500 ml of deionized water. Each tray was treated with different volumes of larval diet (100% vol/vol) as shown in the Table 1.

The larval diet mentioned in Table 1 was provided twice a day (morning and evening) until pupation. Excessive food, fecal matter, and debris in the larval trays were removed daily using pasture pipettes, in order to maintain satisfactory water quality levels for larval development. All larval trays were examined daily at 09:00 and 15:00 h, and pupae that had formed were manually collected. The pupae were counted and placed in transparent plastic vials (50 ml capacity) filled with 25 ml of deionized water and were covered with a sponge plug. Adult emergence was also recorded daily, and sex was determined. The experimental setup was repeated five times.

### 2.3. Morphometric Characterization of Larvae Reared under Different Food Concentrations

Randomly selected five larvae were preserved daily from each diet treatment. Morphometric parameters, namely, head length, head width, thoracic length, thoracic width, abdominal length, abdominal width, and total length of larvae were measured using a stereo microscope (BOECO BST-606, Germany) fixed with a microscopy digital USB camera (Optika 4083. B6) and OPTIKA version 2.12 image processing software.

### 2.4. Pupation Success and Size Variation of Pupae

Number of emerged pupae and mortality were recorded daily. The pupation success (percentage of larvae that emerged as pupae) was calculated at each dietary concentration [20]. Five pupae from each diet treatment were randomly selected, and selected morphometric characteristics (length and width of the cephalothorax) were measured using the above mentioned stereo microscope.

### 2.5. Adult Emergence Rate, Oviposition Rate, and Adult Longevity

Emerged adult mosquitoes from each diet treatment were collected and counted. The adult emergence rate was calculated as the percentage of adult mosquitoes emerged from the total number of pupae under each treatment. The sex of the mosquitoes was determined and recorded while transferring to new separated cages provided with 10% sugar and vitamin B syrup. Females were allowed to feed on human blood using an artificial membrane feeding system 24 hours after sugar feeding [19]. Egg laying cups for females were placed inside cages in order to facilitate the oviposition. The number of eggs in the filter paper were counted every day and replaced with new lining until the colony was over. The durations of adult survival were counted as the longevity of adults [20].

### 2.6. Size Discrimination of Adult Mosquitoes

Five adult males and females that emerged from each diet treatment were collected randomly into separate collection tubes using mouth aspirators and sacrificed with a cold shock. Wings were separated with a dissecting microscope. The standardized wing length was measured from the distal end of alula to the end of radius vein, excluding fringe scale [18], and the width of the wing was measured at the greatest breadth excluding fringing scales. In addition, measurements were made for thorax width from dorsum to coxae and from the base of neck to base of the abdomen. Abdomen width was measured at the widest point, and the length of the abdomen was measured from base to the tip.

All measurements were taken by using a stereo microscope (BOECO BST-606, Germany) fixed with a microscopy digital USB camera (Optika 4083. B6) and OPTIKA version 2.12 image processing software. The digital images of wing, thorax, and abdomen were made by digital camera mounted stereo microscope with the same magnification (8x).

### 2.7. Survival Rates of Larvae, Pupae, and Adults at Different Food Concentrations

The number of surviving larvae, pupae, and adult mosquitoes were observed and recorded twice a day, separately. The survival rate of *Ae*. *aegypti* was calculated as the proportion of larvae, pupae, or adults that survived the relevant period in the life cycle over the total number of individuals that were present in the considering life stage at the beginning [18].

### 2.8. Effect of Larval Food Quantity on Biting Frequency

A total of 20 newly emerged adult mosquitoes from each diet treatment was transferred into a new cage with 1 : 1 sex ratio. A blood meal of human origin was provided artificially for 10-minute duration in the arms of a human host (a healthy volunteer). The proportion of females that ingested or attempted to ingest blood was observed by three well-trained technical staff members. The biting frequency was estimated as the percentage of females that ingested or attempted to ingest blood over the total number of female mosquitoes [21, 22]. The experiment was repeated for 5 times.

### 2.9. Determination of the Vectorial Capacity of *Ae*. *aegypti*

Vectorial capacity (VC) of *Ae*. *aegypti* treated with different diet concentrations was calculated according to the below mathematical approximation [21]:

(1) $$\begin{array}{c} \text{VC}=\frac{ma^{2}p^{n}}{-\log\;eP}, \end{array}$$

where **m** = vector density, **a** = average biting frequency, **p** = survivorship, and **n** = extrinsic incubation period [21]. The vector density was considered as 100 females since 200 *Ae*. *aegypti* mosquitoes (with 1 : 1 male : female ratio) were maintained in a single case for under different treatments. The daily survival rate *P* was calculated using a mathematical formula $\sqrt[{d}]{P}$, where *d* is the duration of study (28 days) and *P* is proportion of females survive by the end of that period [23]. The mean extrinsic incubation period for dengue virus in *Ae*. *aegypti* was considered as 6.5 days at 30°C (best fitting value for Sri Lankan conditions) as recommended by Chan and Johansson [24].

### 2.10. Data Processing and Statistical Analysis

All the data were entered into Microsoft Excel Work Sheet and were analyzed using IBM SPSS Statistics (version 23 copyright IBM Corporation). The effect of larval diet concentration on mortality rate, survival rate of larvae, survival rate of pupae, survival rate of adult, pupation success, oviposition rate, longevity of adults, size discrimination of larvae, size discrimination pupae, and size discrimination of adults was analyzed using General Linear Model (GLM), followed by means separation by Tukey's HSD (honest significant difference) at 5% level of significance. The effect of larval diet concentration on the vectorial capacity and biting frequency was also analyzed using GLM.

## 3. Results

### 3.1. Larval Stage

#### 3.1.1. Effect of Larval Diet on Larval Growth and Survival

Larval food treatment significantly affected the larval growth and survival to pupation. Head length (*F*_4,14_ = 29.599; *P* < 0.001), head width (*F*_4,14_ = 20.797; *P* < 0.001), thoracic width (*F*_4,14_ = 55.924; *P* < 0.001), abdominal length (*F*_4,14_ = 10.381; *P* < 0.001), abdominal width (*F*_4,14_ = 7.239; *P*=0.001), and total length (*F*_4,14_ = 20.797; *P* < 0.001) of the 4^th^ instar *Ae*. *aegypti* larvae denoted significant variations with different larval diet concentrate as indicated by the General Liner Model (GLM) at 5% degree of significance (Table 2).

Larvae reared under 10% larval diet treatment had the highest head length (0.66 ± 0.20 mm), head width (0.64 ± 0.20 mm), thoracic width (0.94 ± 0.0 mm), abdominal length (3.74 ± 0.20 mm), abdominal width (0.48 ± 0.04 mm), and total length (4.95 ± 0.20 mm). Interestingly, even though the variation of thoracic length of 4^th^ instar was not significant (*F*_4,14_ = 2.136; *P*=0.127), the highest thoracic length of 0.673 ± 0.50 mm was observed from the larvae fed with 10% larval diet concentration. The lowest values for all the morphological features of 4^th^ instar larvae were observed at 6% larval diet concentration.

**Figure 1:**
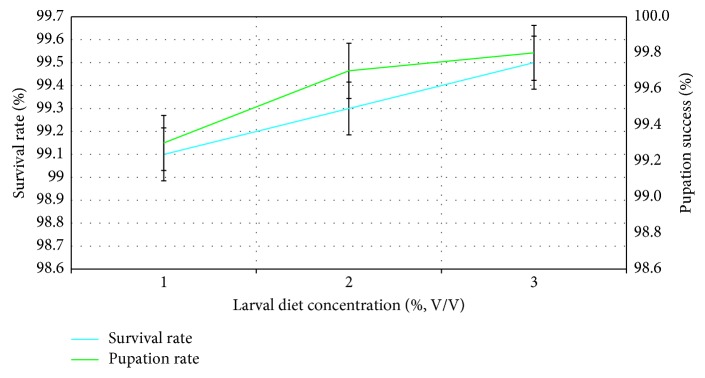
Effect of larval diet concentration (%, V/V) on survival rate (%) and pupation success (%) of larvae of *Ae*. *aegypti*.

Even though survival rates of larvae gradually increased with the ascending larval diet concentrations, whereby the highest survival rate (99.5 ± 0.20%) was observed at 10% larval diet concentration, the survival rates of larvae also did not vary significantly (*F*_4,14_ = 0.230; *P*=0.104) as indicated in Figure 1.

### 3.2. Pupal Stage

#### 3.2.1. Effect of Larval Diet Concentration on Pupation Success, Pupal Morphometry and Survival and Adult Emersion Rates

**Figure 2:**
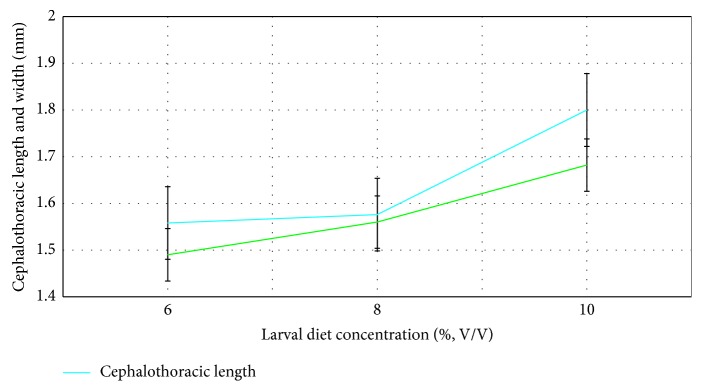
Effect of larval diet concentration (%, V/V) on cephalothoracic length (mm) & cephalothoracic width (mm) of pupae of *Ae*. *aegypti*.

The pupation success was not significantly associated with the larval diet concentration (*F*_4,14_ = 0.027; *P*=0.973) and the pupae originated from the larvae treated with 10% larval diet had the highest pupation success of 97.80 ± 0.2% (Figure 1). The highest cephalothoracic length (1.80 ± 0.10 mm) and cephalothoracic width (1.80 ± 0.10 mm) of pupae were noted from the 10% larval diet concentration, while 6% larval diet concentration denoted the lowest. However, the variation of cephalothoracic length (*F*_4_,_14_ = 0.907; *P*=0.416) and cephalothoracic width (*F*_4_,_14_ = 2.777; *P*=0.08) with the larval diet level were not significant (Figure 2).

**Figure 3:**
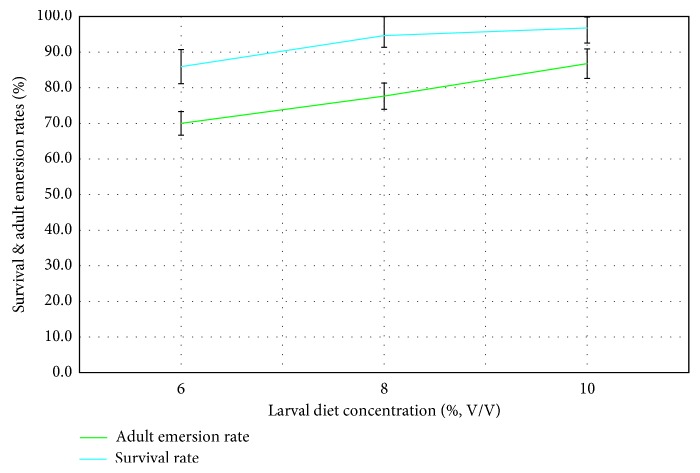
Effect of larval diet concentration (%, V/V) on survival rate (%) and adult emersion rate (%) of pupae of *Ae*. *aegypti*.

The survival rate of pupae indicated a significant gradual boost with increasing concentration of larval diet, whereby the highest survival rate of pupae (99.50 ± 0.02%) was observed at 10% larval diet concentration, while the lowest (89.10 ± 0.05%) was indicated at 6% concentration level (Figure 3), which remained statistically significant according to GLM (*F*_4,14_ = 24.011; *P*=0.014).

### 3.3. Adult Stage

#### 3.3.1. Effect of Larval Diet Concentration on Adult Emersion Rate and Adult Morphometry

The highest adult emersion rate of 86.5 ± 2.8% was observed from the 10% larval diet dose, while the 6% treatment reported the lowest (69.0 ± 3.8%) as denoted in Figure 3. According to GLM, the adult emersion rates (*F*_4,14_ = 54.14; *P* < 0.001) of pupae of *Ae*. *aegypti* treated with different larval diet concentrations varied significantly. Meanwhile, the mosquito larvae that were subjected to low-diet treatment during larval stages were significantly smaller than that were reared under low diet levels. The thoracic length (*F*_4,14_ = 4.662; *P*=0.014), abdominal length (*F*_4_,_14_ = 12.452; *P*=0.001), abdominal width (*F*_4,14_ = 5.890; *P*=0.05), and wing length (*F*_4,14_ = 7.001; *P*=0.02) of adult of *Ae*. *aegypti* were significantly affected by varying diet concentrations.

Meanwhile, the thoracic width of adults did not show any significant variances (*F*_4_,_14_ = 2.469; *P*=0.094) at different diet concentrations (Table 3). On the other hand, the effect of gender on all the above morphometric parameters was significant (*P* < 0.05), except for thoracic length (*F*_4_,_14_ = 47.38; *P*=0.184). However, the highest thoracic length (1.52 ± 0.03 mm), thoracic width (1.03 ± 0.02 mm), abdominal length (2.83 ± 0.03 mm), abdominal width (0.75 ± 0.01 mm), and wing length (3.36 ± 0.03 mm) were observed from female mosquitoes of *Ae*. *aegypti* developed under the 10% larval diet concentration (Table 3).

#### 3.3.2. Impact of Larval Diet on Functional and Behavioral Characteristics of Adult *Ae*. *aegypti*

Rearing under different larval diet doses resulted in significant variations in biting frequency (*F*_4,14_ = 47.50; *P* < 0.001), fecundity (*F*_4,14_ = 18.33; *P*=0.012), and survival rate (*F*_4,14_ = 3.61; *P*=0.016) of adult female mosquitoes of *Ae*. *aegypti*. Interstingly, the longevity of adults of *Ae*. *aegypti* (*F*_4,14_ = 0. 591; *P*=0.608) did not indicate any significant variations with the different larval diet concentrations in accordance with the GLM. Even though longevity was not significantly affected by larval diet, females treated under 10% diet were detected with highest longevity (37.0 ± 2.0 days). Females emerged from larvae treated with low-diet concentrations produced significantly fewer eggs than females provided with high level of food. In addition, the highest biting frequency (99.60 ± 0.40%) and survival rate (97.95 ± 0.65%) of larvae were observed at larvae treated with 10% diet (Table 4).

#### 3.3.3. Vectorial Capacity of Adult *Ae*. *aegypti*

**Figure 4:**
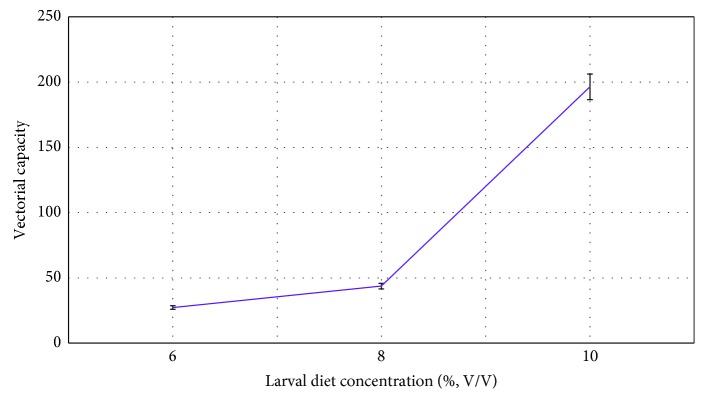
Effect of larval diet concentration (%, V/V) on vectorial capacity of adult *Ae*. *aegypti*.

Adults reared under the highest dietary treatment (10%), were characterized with the highest VC (196.37 ± 29.92) followed by 8% larval diet treatment with (27.30 ± 4.47). Results of GLM indicated that VC of adult *Ae*. *aegypti* differed significantly (*F*_4,14_ = 24.048; *P*=0.014, at 95% level of confidence) among the different larval diet concentrations (Figure 4). Similar to the previously observed trends, adults produced from the two trays with 8% and 6% concentrated diets formed one subset (a), while adults from the highest concentrated diet (10%) formed the other subset (b) in terms of VC.

## 4. Discussion

Vectorial capacity (VC) is the capability of a vector to transmit pathogens to a host resulting in a vector-borne disease [18]. It is an important index to predict epidemiological consequences of any entomological change, as vectorial capacity is thus a density-dependent attribute of the mosquito influenced by behavioral, ecological, and environmental factors [25]. Therefore, evaluation of the vectorial capacity of disease vectors is an essential biological parameter to design control strategies in vector control programmes.

The present study investigated the effect of larval diet concentration on life history traits and VC of *Ae*. *aeygpti*, which is the main vector for dengue disease transmission. There are several advantages in gathering information related to larval biology, which can be ultimately used in modelling, forecasting population dynamics [26], and improving vector control measures [27]. In addition, it can also be used to improve the existing *Ae*. *aegypti* rearing procedures under laboratory in order to promote faster larval development, higher survival rates, and production of homogeneous adult population targeting the release of transgenic or sterile mosquitoes as a vector control measure in the integrated vector management.

According to previous research findings, larval food availability severely influences the growth of larvae and results smaller-sized larvae under low level of larval diet. However, food availability is mostly important in 4^th^ instar larval stage before pupation than first three larval instar stages [28]. Current study revealed that the highest larval growth was examined in the larval diet treatment with 10% highest concentration. Yet, lowest larval growth was shown by the larvae reared in the 6% treatment with minimum larval food amount. According to the statistical analysis, most of larval growth parameters varied significantly with different larval dietary concentrations including head length, head width, thoracic width, abdominal length, abdominal width, and total length of larvae.

Only thoracic length did not show a significant difference at different larval diet concentrations. However, life history traits in terms of larval development, female size, and fecundity are affected by larval nutritional stress. Generally, larvae reared under limited food availability conditions need more time to develop and less likely to be gravid than treated with high food [13]. On the other hand, the larval competition for food and other means may enhance dengue viral susceptibility and diminish physiological hindrances to dissemination and transmission, since it could reduce virulence of virus by reducing the force of selection [10].

According to previous studies, sometimes excessive larval diet can reduce larval survival due to the presence of microorganisms, which proliferates unconsumed food availability resulting larval mortality [29]. As investigated by Puggioli et al., the larval development can be either positively or negatively affected depending on the microbial colony, providing additional nutrients to larvae or contaminating feed and make it inedible to larvae [18]. Moreover, according to Lara, poor diet causes an extended larval period, and since immature spend 25% of their biomass on average moulting, mosquito larvae must acquire enough food supply for ecdysis to avoid a high mortality rate [30].

Some recent studies have indicated that the fitness and health status of adult mosquitoes can be estimated in indirectly by referring to the body size [31]. Significant associations were observed between total length and thoracic length with larval diet levels. In addition to larval growth parameters, cephalothoracic length, and width of pupae varied significantly with larval diet level [31].

In this investigation pupal development was also measured as cephalothoracic length and cephalothoracic width. However, no significant difference was observed between larval diet concentrations with both cephalothoracic length and cephalothoracic width as indicated by the results of GLM. Probably the reason for the above results may be the random and unbiased sampling of pupae for the experimental procedure in the pupal measurements.

The lowest survival rate (89.1 ± 0.05%) was shown by pupae at the lowest larval diet treatment. According to the statistical analysis, the survival of pupae significantly increased with increasing food amounts, probably due to nutrition reserves rich in lipids and that accumulated during the larval stages [32, 33]. This may account for the high mortality observed from pupae subjected to low food supply. Current study revealed that highest pupation success of larvae was observed at highest larval diet concentration (10%), and larvae reared under lowest larval diet level (6%) were characterized with the lowest pupation success. Previous investigations have shown that pupation success (ability of larvae to pupate) is greatly influenced by the nutritional reserves acquired during feeding [34]. According to Chambers and Klowden, mosquito larvae have to achieve a critical weight in order to pupate, allowing the larvae reared with higher food supply probably attain their critical weights earlier than those reared under lower food supply [35].

In addition to the larval and pupal growth, adult body parts are also measured including thoracic length, thoracic width, abdominal length, abdominal width and wing length to study about growth of adult. In accordance with Petersen et al., the measurement of adult body parts also can be used to evaluate the fitness and health of adult mosquitoes indirectly [35].

Findings of the study revealed that the effect of larval diet concentrations on most of measurements of both adult males and females remained significant except for the thoracic width. In fact, the effect of gender on the adult measurements also remained significant. The largest males and females were obtained with highest concentrated diet treatment (10%) and adults undergo reduction of body size with lowering the concentration of larval diet.

Biting frequency of *Ae*. *aegypti* has affected a number of factors such as necessity of intaking small blood meals [36], need to increase fecundity [37], and need to gain additional nutrition to survive in stressful environments [38] and due to host availability. Biting frequency of adult females significantly varied with different larval diet concentrations in accordance with the results of the present study. Adults reared at the highest concentrated diet (10%) showed the highest biting frequency (99.6 ± 0.40%), while adults reared at the lowest concentrated diet (6%) showed the lowest biting frequency (76.56 ± 1.52%).

The group of mosquitoes fed with higher food amounts had relatively higher longevity than adults that emerged from larvae reared within a low food environment. However, the longevity of adults was not affected significantly by the food amount available during the larval stages in the present study. As suggested by previous studies, nutrient reserves of adults obtained during the larval stage contribute to an increased the longevity [11], which may explain the shorter longevity of adults derived from the larvae supplied with lower food amount.

Larvae supplied with the higher food amounts resulted adults with higher fecundity and higher survival rate. The amount of food availability to the larvae significantly affected the number of eggs oviposited per female (fecundity) and their survival rate. Current study revealed that the highest VC (196.37 ± 29.92) was observed at highest larval diet concentration (10%), and larvae reared under lowest larval diet level (6%) were characterized with the lowest vectorial capacity (27.30 ± 4.47).

According to previous research findings, smaller females often require two to three blood meals to develop their first batch of eggs and biting frequency may increase the probability of smaller females in acquiring an infectious blood meal [39]. In addition, longer blood meals by larger females may increase the number of parasites ingested, thereby increasing the probability of capturing the infection by the vector [39, 40]. In malaria vectors, the size of the mosquito also influences oocyst numbers in the midgut of naturally infected mosquitoes, supporting the hypothesis that mosquitoes arising from well-nourished larvae are more competent for parasite transmission [41].

In dengue vectors, some studies have hypothesized that the smaller mosquitoes emerged from high-density treatments with low food availability may enhance susceptibility to dengue virus as smaller mosquitoes may have reduced immune functions [42] and reduced physiological barriers to viral dissemination [43]. However, increase in viral dissemination with time may not directly contribute to enhance VC, since cumulative effect of adult mortality and increase in viral dissemination rate which present at latter incubation period may more unlikely affect VC [10].

The present study did not investigate the vector competence in adult mosquitoes due to limitations in resources and facilities. However, for calculation of vectorial capacity, information on the extrinsic incubation was considered from previously published literature. Current study reported a more than 5-fold increment in the vectorial capacity of *Ae*. *aegypti*, relative to the larvae treated under lowest concentration of diet. Therefore, limitations in larval food availability could have significant biological implications on the vector bionomics of *Aedes* mosquitoes in the natural environment.

According to the findings of some investigations, it is stated that the larval competition in terms of space or food may result viral strains with shorter extrinsic incubation period in mosquitoes [44]. This phenomenon may also adversely influence on human health since dengue virus isolates with short extrinsic incubation period in mosquitoes are associated with more severe clinical symptoms in humans [45].

## 5. Conclusions

It can be concluded that multiple biological parameters of *Ae*. *aegypti* are significantly affected by the amount food provided to larvae. The larvae fed with higher food amounts produced adults with larger wing sizes and longer longevity, greatly increasing the vectorial capacity. Therefore, difference in the quality of larval habitats could have crucial implications for dynamics of dengue transmission. As there are several interplaying factors that affect the viral transmission in the natural environment, these aspects should be further investigated though multifactorial experiments. The present study may also provide essential information for colonization of *Ae*. *aegypti* mosquitoes in large scale mass rearing for open release in order to achieve vector reduction by population suppression or replacement strategies.

## Figures and Tables

**Table 1:** Composition of larval diet in different concentration and feeding arrangement.

Tray no.	Larval diet volume introduced (ml)	Diet concentration (vol/vol) (%)	No. of larvae at one trial (*n*)
1	0.25	10	400
2	0.20	8	400
3	0.15	6	400

**Table 2:** Measurements of 4^th^ instar larvae *Aedes aegypti* fed with different concentration of larval diet.

Larval diet concentration (%)	Head length (mm)	Head width (mm)	Thoracic length (mm)	Thoracic width (mm)	Abdominal length (mm)	Abdominal width (mm)	Total length (mm)
6	0.51 ± 0.20^a^	0.53 ± 0.20^a^	0.56 ± 0.50^a^	0.55 ± 0.04^a^	2.85 ± 0.20^a^	0.31 ± 0.04^a^	3.90 ± 0.20^a^
	(0.31–0.71)	(0.33–0.73)	(1.06–0.06)	(0.51–0.59)	(2.65–3.05)	(0.27–0.35)	(3.70–4.10)
8	0.56 ± 0.20^b^	0.54 ± 0.20^a^	0.58 ± 0.50^a^	0.59 ± 0.04^a^	3.33 ± 0.20^b^	0.39 ± 0.04^a,b^	4.35 ± 0.20^a^
	(0.36–0.76)	(0.34–0.74)	(1.08–0.08)	(0.55–0.63)	(3.13–3.53)	(0.35–0.43)	(4.15–4.55)
10	0.66 ± 0.20^c^	0.64 ± 0.20^b^	0.67 ± 0.50^a^	(0.94 ± 0.04)^b^	3.74 ± 0.20^b^	0.48 ± 0.04^b^	4.95 ± 0.20^b^
	(0.46–0.86)	(0.44–0.84)	(1.17–0.17)	(0.90–0.98)	(3.54–3.94)	(0.44–0.52)	(5.15–4.75)

*Note*. Values are mean ± SE, range in parenthesis. Different superscript letters in a column show significant differences (*P* < 0.05) as suggested by General Linear Modelling followed by Tukey's HSD (honest significant difference) at 95% level of significance.

**Table 3:** Measurements of adult *Aedes aegypti* resulted from different larval nutritional regimes.

Larval diet concentration (%)	Thoracic length (mm)		Thoracic width (mm)		Abdominal length (mm)		Abdominal width (mm)		Wing length (mm)	
	Male	Female	Male	Female	Male	Female	Male	Female	Male	Female
6	1.00 ± 0.04^a^	1.34 ± 0.03^a^	0.75 ± 0.02^a^	0.97 ± 0.02^a^	2.20 ± 0.03^a^	2.60 ± 0.03^a^	0.44 ± 0.02^a^	0.64 ± 0.01^a^	2.36 ± 0.04^a^	3.12 ± 0.03^a^
	(0.06–1.04)	(1.31–1.37)	(0.73–0.77)	(0.95–0.99)	(2.17–2.23)	(2.57–2.63)	(0.42–0.46)	(0.63–0.65)	(2.32–2.40)	(3.09–3.15)
8	0.99 ± 0.04^a^	1.33 ± 0.03^a^	0.74 ± 0.02^a^	0.96 ± 0.02^a^	2.21 ± 0.03^a^	2.60 ± 0.03^a^	0.47 ± 0.02^a,b^	0.68 ± 0.01^a,b^	2.37 ± 0.04^a^	3.13 ± 0.03^a^
	(0.95–1.03)	(1.30–1.36)	(0.72–0.76)	(0.94–0.98)	(2.18–2.24)	(2.58–2.63)	(0.45–0.49)	(0.67–0.69)	(2.33–2.41)	(3.10–3.16)
10	1.14 ± 0.04^b^	1.52 ± 0.03^b^	0.80 ± 0.02^a^	1.03 ± 0.02^a^	2.40 ± 0.03^b^	2.83 ± 0.03^b^	0.51 ± 0.02^b^	0.75 ± 0.01^b^	2.54 ± 0.04^b^	3.36 ± 0.03^b^
	(1.10–1.18)	(1.49–1.55)	(0.78–0.82)	(1.01–1.05)	(2.37–2.43)	(2.80–2.86)	(0.49–0.53)	(0.74–0.76)	(2.50–2.58)	(3.33–3.39)

*Note*. Values are mean ± SE, range in parenthesis. Different superscript letters in a column show significant differences (*P* < 0.05) as suggested by General Linear Modelling followed by Tukey's HSD (honest significant difference) at 95% level of significance.

**Table 4:** Biting frequency, longevity, fecundity, and survival rate of adult *Aedes aegypti* fed with different concentration of larval diet.

Diet concentration (%)	Biting frequency (%)	Longevity (days)	Fecundity (number of eggs)	Survival rate (%)
6	76.56 ± 1.52^a^	33.5 ± 2.5^a^	60.4 ± 10.8^a^	94.95 ± 0.35^a^
	(75.04–78.08)	(31.0–36.0)	(49.6–71.2)	(94.60–95.30)
8	82.91 ± 3.07^a^	34.50 ± 2.5^a^	65.24 ± 6.32^a^	95.3 ± 1.30^b^
	(82.84–82.98)	(32.0–37.0)	(58.92–71.56)	(94.0–96.6)
10	99.6 ± 0.4^b^	37.0 ± 2.0^a^	116.40 ± 0.78^b^	97.95 ± 0.65^b^
	(99.2–100.0)	(35.0–39.0)	(115.62–117.18)	(97.30–98.60)

*Note*. Values are mean ± SE, range in parenthesis. Different superscript letters in a column show significant differences (*P* < 0.05) as suggested by General Linear Modelling followed by Tukey's HSD (honest significant difference) at 95% level of significance.

## Data Availability

The data used to support the findings of this study are available from the corresponding author upon request.
